# Headache among children and adolescents in Benin: revised prevalence estimates, and estimates of attributed burden, from a national schools-based study

**DOI:** 10.1186/s10194-026-02448-2

**Published:** 2026-07-16

**Authors:** Mendinatou Agbetou Houessou, Thierry Adoukonou, Willy Tchuenga Fokom, Nelly Dovoedo, Tayyar Şaşmaz, Fatma Bozdağ, Derya Uluduz, Andreas Kattem Husøy, Timothy J. Steiner

**Affiliations:** 1 Neurology Department, University of Parakou, Parakou, Benin; 2 National Teaching Hospital HKM of Cotonou, University of Abomey- Calavi, Cotonou, Benin; 3 Department of Public Health, Mersin University School of Medicine, Mersin, Turkey; 4 Division of Occupational Medicine, Department of Public Health, Faculty of Medicine, Gazi University, Ankara, Turkey; 5 Department of Neurology, Faculty of Medicine, Istanbul Atlas University, Istanbul, Turkey; 6 NorHead, Department of Neuromedicine and Movement Science, Norwegian University of Science and Technology, Edvard Griegs Gate, Trondheim, Norway; 7 Department of Neurology and Clinical Neurophysiology, St Olavs University Hospital, Trondheim, Norway; 8 Department of Neurology, University of Copenhagen, Copenhagen, Denmark; 9 Department of Brain Sciences, Imperial College London, London, UK

**Keywords:** Child and adolescent headache, Migraine, Tension-type headache, Medication-overuse headache, Undifferentiated headache, Epidemiology, Burden of disease, Schools-based study, Benin, Sub-Saharan Africa, Global Campaign against Headache

## Abstract

**Background:**

In our earlier report of headache prevalence among children (6–11 years) and adolescents (12–17) in Benin, undifferentiated headache (UdH) was defined conventionally as mild headache lasting < 1 h. In this and similar studies elsewhere in sub-Saharan Africa, we recognised diagnostic uncertainties among the many participants with short-duration headache. Here we make new estimates of prevalence using a modified definition of UdH, and, based on these, estimates of attributed burden. The study was part of the global schools-based programme conducted by the Global Campaign against Headache.

**Methods:**

In a cross-sectional survey, the child and adolescent versions of the structured HARDSHIP questionnaire, translated into West-African French, were completed by pupils in class in 16 schools representative of Benin’s urban/rural divide. Headache diagnoses were based on ICHD-3 except for UdH, which we redefined as *mild or moderate* headache lasting < 1 h. Enquiry included multiple aspects of attributed burden, with a timeframe of 4 weeks and additional enquiry into headache yesterday (HY).

**Results:**

Of 2,488 eligible pupils, 193 (or their parents) declined, while 2,295 completed the questionnaires (males 1,156 [50.4%], females 1,139 [49.6%]; children 1,081 [47.1%], adolescents 1,214 [52.9%]; mean age 12.1 ± 3.1 years [median 12]; participating proportion 92.2%). With the modified definition, observed 1-year prevalence of UdH increased from 8.4% to 21.0%, with corresponding reductions for migraine (from 52.1% to 44.9%) and tension-type headache (TTH) (from 21.8% to 17.2%). Mean symptom burden was expressed in moderate headache on 4.2 days/4 weeks, with duration 2.8 h. All headache accounted for 4.0% lost school time according to 4-week recall, 3.3% when assessed from HY (which could not capture lost days immediately preceding weekends or holidays). Almost one in three parents (30.3%) lost time from their own work, those of children more than those of adolescents. Measures of impaired participation clearly distinguished migraine (greater burden) from TTH and UdH

**Conclusions:**

Headache is prevalent among children and adolescents in Benin. The associated lost health and impaired participation, including lost school time with, potentially, lifelong negative impact, are reliably attributed to headache. Attribution to specific headache types is less certain because of unresolved diagnostic difficulties surrounding short-duration headache.

## Introduction

We have previously reported headache prevalence among children (6–11 years) and adolescents (12–17) in Benin [1], a Francophone country in western sub-Saharan Africa (SSA). In this report, undifferentiated headache (UdH) was defined conventionally as mild headache of duration < 1 h [2]. Here we adopt modified criteria for UdH, and revise the prevalence estimates accordingly. We also report estimates of headache-attributed burden.

UdH was first identified in a study in Turkey [2], where the methodology was developed [3] for the global schools-based programme of burden-of-headache studies among children and adolescents conducted by the Global Campaign against Headache [4, 5]. In this study, many participating pupils reported mild headache of short duration (< 1 h) [2], not meeting any set of criteria of the International Classification of Headache Disorders (ICHD) [6]. Subsequent studies in this programme, in Austria [7], Lithuania [8], Georgia [9], Iran [10], Nepal [11] and Mongolia [12], found the same. We believed that these headaches were expressions by the immature brain of what would later develop into migraine or tension-type headache (TTH) [2].

In studies in SSA, our 1-year prevalence estimates for migraine (definite + probable) were unfeasibly high: in Ethiopia 38.6% [13], in Zambia 53.2% [14] and here in Benin 53.4% [1]. Outside SSA, in Nepal in South East Asia, we initially found an even higher prevalence (58.4% [11]). We recognised that the difference between “mild” and “moderate” headache was very often the driver of diagnostic distinction between UdH and probable migraine, and that this distinction was very highly subjective, especially among children. We argued that it ought not to be the determining criterion, that short duration was the crucial feature of UdH, and that the criteria for UdH should be modified to *mild or moderate headache*, still of duration < 1 h [9, 11, 15].

In the diagnostic hierarchy, UdH is diagnosed after headache disorders characterised by headache on ≥ 15 days/month (H15+), which include probable medication-overuse headache (pMOH), but before migraine or TTH. Modifying the criteria for UdH changes the prevalence estimates for probable migraine and for definite and probable TTH, requiring the revised estimates presented here.

Estimates of burden attributed to headache overall and to each headache type, also presented here across multiple domains, are based on these revised prevalence estimates. Burden estimates are more informative to health and education policies in Benin than those of prevalence. However, all contribute to the Global Burden of Disease (GBD) study [16], to our understanding of headache among these age groups, and to our knowledge of the global burden of headache disorders.

## Methods

This was a schools-based cross-sectional survey. Other than with regard to the definition of UdH, the detailed methodology has been published previously [1, 3], so we present only a summary here.

The study was conducted from April to June 2019, with burden data collected at the same time and from the same participants as the prevalence data.

### Ethics and approvals

This research was performed in accordance with the Declaration of Helsinki.

The study was approved by the Ethics Committee of the University of Parakou (ref: 0265/CLERB-UP/P/SP/R/SA).

The survey was explained to school managers and teachers of the selected schools, whose agreement to participate was necessary. Information forms describing the nature and purposes of the study were given to all intended participants, for themselves and their parents. Pupils’ consents were required for participation. In keeping with the terms of ethics approval, parents were asked to notify the school should they object, and were presumed to be in agreement if they did not.

Data were collected anonymously, and data protection legislation was complied with.

### Study procedures

We used the standardised protocol for the global series [3]. We selected 16 schools in three of the country’s four departments aiming for urban/rural and public/private parity with respect to the country as a whole. We invited all pupils aged 6–17 years, from all classes, who were present on the survey days and for whom no parental objection had been registered, to participate. We used the child and adolescent versions of the structured Headache-Attributed Restriction, Disability, Social Handicap and Impaired Participation (HARDSHIP) questionnaire [3], translated into West African French according to LTB’s translation protocol for hybrid documents [17]. These were completed by pupils in mediated sessions within their classes.

HARDSHIP included demographic enquiry and headache diagnostic questions based on ICHD-3 [6] other than for UdH. Burden enquiry included symptom burden, medication use, headache-attributed lost health, impaired participation (lost time from school and other activities), lost parental worktime, emotional impact and quality of life (QoL). The timeframes of enquiry were 4 weeks and 1 day, the latter based on headache yesterday (HY).

We performed independent double data-entry, with reconciliation by reference to the source data [1].

### Analysis

Initial analyses, using the conventional definition of UdH, were performed at University of Mersin, Turkey. Subsequent analyses, applying the modified definition, were performed at the Norwegian Centre for Headache Research (NorHead).

In the burden analyses, we combined definite and probable migraine and definite and probable TTH.

We recorded headache frequency and acute medication intake as days/4 weeks. Headache intensity was reported as “mild”, “moderate” or “severe”, which we converted to a numerical scale of 1–3, treating this as continuous. Duration of episodes was reported in hours as < 1, 1–2, 2–4 or > 4, which we converted for analysis by taking the mid-points (0.5, 1.5, 3 and 8 respectively, assuming a range of 4–12 h for the last). We calculated proportion (%) of time in ictal state (pTIS) from frequency and duration. We estimated lost health at individual level (%) as pTIS*DW, where DW, on a scale 0–1, was the disability weight attributed in the Global Burden of Disease (GBD) study to the ictal state of the headache type (migraine, TTH or pMOH; 0 indicating no health loss and 1 a health loss equivalent to being dead) [18]. We estimated lost health at population level by factoring in prevalence.

We made prevalence estimates as proportions (%), and adjusted observed values for age and gender using population statistics for Benin (in 2018) from PopulationPyramid [19].

We counted days of lost school time because of headache in the preceding 4 weeks. We considered not going to school as a whole day lost, and leaving school early as a half-day lost, and calculated proportions using a denominator of 20. We separately counted days of limited activity (defined as “could not do things you wanted to because of your headaches”), with a presumed denominator of 28. We counted parents of participating pupils who had, according to the pupils, lost time from their own work (regardless of quantity) during the same period. We calculated proportions (%) reporting a lost school day yesterday because of HY, and predicted values of lost school yesterday from the product of number affected by headache and mean reported days (divided by 20) lost per pupil over the preceding 4 weeks. Responses to questions addressing concentration, emotional impact and QoL were all on a 4-point Likert scale (“never”, “sometimes”, “often”, “always”) [3], also referring to the preceding 4 weeks. We scored these 0–3, and summed them to generate an emotional impact score (including concentration; potential range 0–18, high being adverse) and a QoL score (0–36, low being adverse) [3].

In descriptive statistics we used means, standard deviations (SDs) and standard errors (SEs) for continuous variables, and proportions (%) with 95% confidence intervals (CIs) for categorical data. We used Kruskal-Wallis with post-hoc Dunn’s test to evaluate group-differences for continuous variables and binary logistic regression to evaluate group-differences for binary outcomes.

Population-based estimates of burden were derived from our revised prevalence estimates, with UdH redefined as mild or moderate headache lasting < 1 h.

In all analyses, participants with missing data were excluded (there was no imputation). We set significance in all analyses at *p* < 0.05.

## Results

### Sample description

Of 2,488 potential participants (eligible pupils present on the day), 193 declined (or their parents registered objections), while 2,295 completed the questionnaires (males 1,156 [50.4%], females 1,139 [49.6%]; children 1,081 [47.1%], adolescents 1,214 [52.9%]; mean age 12.1 ± 3.1 years [median 12]; participating proportion 92.2% [2,295/2,488]) [1].

### Revised prevalence estimates

Observed 1-year prevalence of any headache was 88.5% (*n* = 2,032) (Table 1), slightly higher among females (90.4%) than males (86.7%; *p* = 0.005), but very similar between children (89.1%) and adolescents (88.1%) (Table 2). HY was reported by 18.7% of participants. These findings have been previously reported [1].

**Table 1:** Observed 1-year prevalence of headache overall and of each headache type according to modified and conventional criteria for undifferentiated headache

Headache type	Modified criteria^1^	Conventional criteria^2^ (from [1])
	% [95% confidence interval]	
Any headache^3^	88.5 [87.2–89.8]	88.5 [87.2–89.8]
UdH	21.0 [19.4–22.7]	8.4 [7.3–9.5]
Migraine	44.9 [42.8–46.9]	52.1 [50.1–54.1]
TTH	17.2 [15.7–18.8]	21.8 [20.1–23.5]
pMOH^3^	1.2 [0.7–1.6]	1.2 [0.7–1.6]
Other H15 + ^3^	2.8 [2.1–3.4]	2.8 [2.1–3.4]
Unclassified	1.4	2.2

UdH: undifferentiated headache, TTH: tension-type headache; pMOH: probable medication-overuse headache; H15+: headache on ≥ 15 days/month; ^1^mild or moderate headache lasting < 1 hour; ^2^mild headache lasting < 1 hour, ^3^these were unaffected by the definition of UdH

Table 1 shows observed prevalence of each headache type under the modified criteria for UdH. For comparison, the table also shows the previously reported values for migraine, TTH and UdH [1], those for pMOH and other H15 + being unaffected. Observed 1-year prevalence of UdH increased from 8.4% to 21.0%. With only a modest reduction, from 52.1% to 44.9%, migraine remained by far the most common headache type, with TTH, reducing from 21.8% to 17.2%, considerably less prevalent in both estimations. H15 + was reported by 4.0%, of whom 1.2% also reported medication overuse (pMOH) (Table 1). Only 1.4% were unclassified with the modified criteria.

Table 2 shows observed prevalence of each headache type by age and gender, along with estimates adjusted for these variables. Significant differences between genders (females > males) were seen only for all headache and other H15+. Between age groups, there were differences for migraine (children > adolescents) and for pMOH, other H15 + and UdH (adolescents > children).

### Burden

Table 3 shows the symptom burden associated with each headache type. Overall headache frequency was 4.2 days/4 weeks, with H15 + inevitably creating a very skewed distribution. Mean overall headache duration was 2.8 h. Mean pTIS was 2.0%. Mean intensity was 2.1 (moderate). Of the episodic headaches, migraine was the most frequent, longest lasting (therefore associated with substantially the highest pTIS) and most intense. Those with pMOH had headache on about 75% of all days, those with other H15 + on rather fewer (about 65%), in each case lasting on average for about 4 h, so that mean pTIS for each of these disorders was about 12% – six times longer than for all headache. pMOH was reportedly as intense as migraine (moderate to severe), other H15 + slightly less (moderate) (Table 3).

Table 4 shows frequency of symptomatic medication use, along with frequency of headache (from Table 3) for comparison. These data reveal that not all headache days were medicated. This is very apparent for other H15+.

Table 5 shows the estimated health loss, corresponding to reductions in healthy life expectancy, associated with each headache type at individual and population levels. These estimates were possible only for those disorders for which DWs had been allocated in GBD (migraine, TTH and pMOH) [18]. At individual level, the health loss attributed to pMOH was substantial (2.7%), much more than was attributed to migraine (1.0%), but at population level, because of its much higher prevalence, migraine accounted for a 16-fold higher loss than pMOH (and more than 50-fold higher than TTH).

Table 6 shows impaired participation (lost time from school and other activities, including playtime) because of headache overall, by headache type and in each age group. All headache accounted for 0.8 days of missed school each 4 weeks, a loss of 4.0% assuming a 5-day week. Migraine (0.8 days lost) had rather greater impact on schooling than TTH or UdH (each 0.6 days lost), whereas pMOH had very serious impact: 4.6 days lost from school equates to 23% of school time, while 5.5 days (reported by adolescents) equates to 27.5%. Days of limited activities were more than days lost from school: 1.4 for all headache, or 5.0% with the presumed denominator of 28 days. pMOH reportedly limited activities on more than one third (10.2/28 = 36.4%) of all days (Table 6).

**Table 2:** Observed 1-year prevalence by headache type, gender and age according to modified criteria for undifferentiated headache, and gender- and age-adjusted prevalence estimates

	All headache (*n* = 2,032)	Migraine (*n* = 1,030)	TTH (*n* = 395)	pMOH (*n* = 27)	Other H15+ (*n* = 64)	UdH (*n* = 483)
**Observed prevalences** (% [95% confidence interval])						
Overall	88.5 [87.2–89.8]	44.9 [42.8–46.9]	17.2 [15.7–18.8]	1.2 [0.7–1.6]	2.8 [2.1–3.4]	21.0 [19.4–22.7]
Gender						
male female	**86.7** [84.7–88.6] **90.4** [88.7–92.1]	44.9 [42.0-47.8] 44.9 [42.0-47.8]	16.5 [14.4–18.7] 17.9 [15.7–20.1]	0.8 [0.3–1.3] 1.6 [0.9–2.3]	**1.7** [1.0-2.5] **3.9** [2.7-5.0]	21.5 [19.2–23.9] 20.5 [18.2–22.9]
Age group (years)						
6–11 12–17	89.1 [87.2–90.9] 88.1 [86.2–89.9]	**50.5** [47.5–53.5] **39.9** [37.1–42.6]	17.4 [15.1–19.7] 17.1 [14.9–19.2]	**0.5** [0.1–0.9] **1.8** [1.1–2.6]	**2.0** [1.2–2.9] **3.5** [2.4–4.5]	**17.3** [15.0-19.6] **24.4** [22.0-26.8]
**Gender- and age-adjusted prevalence estimates** (% [95% confidence interval])						
Overall	89.0 [87.7–90.3]	46.6 [44.5–48.6]	17.0 [15.5–18.6]	1.2 [0.8–1.7]	2.9 [2.3–3.7]	19.9 [18.3–21.7]

TTH: tension-type headache; pMOH: probable medication-overuse headache; H15+: headache on ≥ 15 days/month; UdH: undifferentiated headache; significantly different values (*p* < 0.05) are emboldened

**Table 3:** Symptom burden by headache type and overall

Headache type	Headache frequency days/4 weeks (mean±SD)	Headache duration hours (mean±SD)	Estimated pTIS % (mean±SE)	Headache intensity scale 1–3^1^ (mean±SD)
**Episodic headaches**				
Migraine	3.8±2.9	3.7±3.1	2.3±0.1	2.4±0.6
Tension-type headache	3.3±2.6	3.0±2.3	1.4±0.1	1.9±0.6
Undifferentiated headache	3.0±2.8	0.5± < 0.1	0.2± < 0.1	1.6±0.5
**Headache on ≥ 15 days/month**				
Probable medication-overuse headache	21.1±4.7	3.8±3.1	12.2±2.0	2.6±0.6
Other headache on ≥ 15 days/month	18.3±3.6	4.2±3.3	11.7±1.2	2.2±0.8
**All headache** ^**2**^	4.2±4.4	2.8±2.9	2.0±0.1	2.1±0.7

SD: standard deviation; SE: standard error; pTIS: proportion (%) of time in ictal state; ^1^equating 1–3 to the reported categories “mild”, “moderate” and “severe”, and treating as continuous data; ^2^includes unclassified headache

**Table 4:** Symptomatic medication use by headache type and overall

Headache type	Pupils responding *n*	Medication use days in preceding 4 weeks (mean ± SD)	Headache frequency days/4 weeks (from Table 3)
**Episodic headaches**			
Migraine	1,030	2.3±2.1	3.8±2.9
Tension-type headache	395	1.8±1.8	3.3±2.6
Undifferentiated headache	483	1.7±2.0	3.0±2.8
**Headache on ≥ 15 days/month**			
Probable medication-overuse headache	27	18.7±4.8	21.1±4.7
Other headache on ≥ 15 days/month	64	5.4±3.6	18.3±3.6
**All headache** ^**1**^	2,032	2.4±2.9	4.2±4.4

^1^Includes unclassified headache

**Table 5:** Headache-attributed lost health by headache type^1^

Headache type	Disability weight (from [18])	Age- and gender-adjusted prevalence (from Table 2) %	Mean pTIS (from Table 3) %	Lost health %	
				Individual^2^ mean	Population^3^
Migraine	0.441	46.6	2.3	1.0	0.48
Tension-type headache	0.037	17.0	1.4	0.05	0.009
Probable medication-overuse headache	0.223	1.2	12.2	2.7	0.03

pTIS: proportion of time in ictal state; ^1^in the Discussion we highlight the unreliability of these estimates; ^2^calculated as the product of mean pTIS and disability weight; ^3^calculated as the product of mean individual lost health and prevalence (with apparent discrepancies due to rounding)

Lost school time was also assessed more objectively from absences yesterday attributed to HY. Table 7 compares these actual counts with predictions based on pupils’ recall of lost days in the past 4 weeks. The findings were variable, with TTH an outlier and too few pupils with pMOH (*n* = 27) to support reliable analysis; for all headache, actual losses were 15.8% *versus* predicted losses of 18.8%. The former, it should be noted, would not have taken account of lost days immediately preceding weekends or school holidays.

Table 8 shows lost parental worktime (of any amount: pupils were not expected to be aware of how much) because of a son’s or daughter’s headache. One in three parents of children (33.0%) lost at least some time in the preceding 4 weeks, rather fewer parents of adolescents (27.8%). This differential was seen for all episodic headache types, but reversed for pMOH and other H15+. Notably, almost three quarters (72.7%) of parents of adolescents with pMOH lost time from their own work.

Table 9 statistically compares the headache types for the impaired participation measures (lost school time, lost time from other activities and lost parental worktime). On the first two measures, TTH and UdH were clearly distinguished from migraine, but not from each other. On the third, UdH was distinguished from migraine but not from TTH. The episodic headaches were distinguished from pMOH on all three measures, but not from other H15+.

Table 10 (which includes similar between-type comparisons) shows emotional impact and QoL scores. With regard to the former, other H15 + was associated with the highest (most adverse) score (9.3/18), differing statistically from all others except pMOH (7.7/18, but *n* = 26), with migraine next worst (8.2). The score for TTH was numerically the same as the score for pMOH, a little higher than that for UdH (7.2). Unfortunately, the protocol requirement to collect QoL data from those with no headache was misunderstood, and this was not done. Of the headache types, H15+ (pMOH and other) and migraine showed greatest impact, all with scores around half (17.5–18.5) of the possible total score of 36. TTH had less impact (20.6), and UdH least (21.3), significantly different from each other and both significantly less than all other headache types.

**Table 6:** Lost time from school and other activities because of headache in the preceding 4 weeks, by headache type and overall, and by age group

Headache type	Headache frequency (from Table 3) days/4 weeks (mean)	Days of lost school	Days of limited activity
		days/4 weeks/affected pupil (mean ± SD)	
**Episodic headaches**			
Migraine	3.8±2.9	0.8±1.3	1.3±1.7
Children Adolescents	4.2±2.8 3.4±2.9	0.9±1.2 0.7±1.4	1.4±1.6 1.3±1.8
Tension-type headache	3.3±2.6	0.6±1.3	1.0±1.5
Children Adolescents	3.7±2.5 2.8±2.5	0.8±1.1 0.5±1.3	1.0±1.3 1.0±1.7
Undifferentiated headache	3.0±2.8	0.6±1.1	1.0±1.7
Children Adolescents	3.9±3.0 2.5±2.6	0.9±1.2 0.4±0.9	1.3±1.9 0.8±1.6
**Headache on ≥ 15 days/month**			
Probable medication-overuse headache	21.1±4.7	4.6±6.3	10.2±10.0
Children Adolescents	20.8±4.4 21.6±4.8	0.5±1.1 5.5±6.6	8.0±9.3 10.7±10.3
Other headache on ≥ 15 days/month	18.3±3.6	1.8±3.1	5.2±6.4
Children Adolescents	18.6±3.3 18.2±3.9	1.4±1.8 2.0±3.6	2.7±5.2 6.5±6.6
**All headache** ^**1**^	4.2±4.4	0.8±1.6	1.4±2.6
Children Adolescents	4.5±3.8 4.0±4.8	0.8±1.2 0.7±1.8	1.3±1.9 1.5±3.1

^1^Includes unclassified headache

**Table 7:** Lost school days yesterday because of headache yesterday (HY), by headache type and overall, with predictions based on recalled lost days/4-week

Diagnosed headache type	Pupils affected *n* (from Table 2)	Proportion reporting HY *n* (% of those with headache type)	Missed school day yesterday *n* (% of those reporting HY)	
			Actual	Predicted
**Episodic headaches**				
Migraine	1,030	216 (21.0)	31 (14.4)	41 (20.0)
Tension-type headache	395	83 (21.0)	19 (22.9)	12 (14.5)
Undifferentiated headache	483	76 (15.7)	11 (14.5)	14 (18.4)
**Headache on ≥ 15 days/month**				
Probable medication-overuse headache	27	18 (66.7)	1 (5.6)	6 (33.3)
Other headache on ≥ 15 days/month	64	30 (46.9)	6 (20.0)	6 (20.0)
**All headache** ^**1**^	2,032	430 (21.2)	68 (15.8)	81 (18.8)

^1^Includes unclassified headache

**Table 8:** Parents’ lost work in the preceding 4 weeks, by pupils’ headache type and overall, and by age group

Headache type	Pupils affected *n* (from Table 2)	Headache frequency days/4 weeks (from Table 3)	Parent missed work *n* (% of pupils with headache type)
**Episodic headaches**			
Migraine	1,030	3.8±2.9	334 (32.4)
Children Adolescents	546 484	4.2±2.8 3.4±2.9	181 (33.2) 153 (31.6)
Tension-type headache	395	3.3±2.6	108 (27.3)
Children Adolescents	188 207	3.7±2.5 2.8±2.5	65 (34.6) 43 (21.3)
Undifferentiated headache	483	3.0±2.8	121 (25.1)
Children Adolescents	187 296	3.9±3.0 2.5±2.6	60 (32.1) 61 (20.6)
**Headache on ≥ 15 days/month**			
Probable medication-overuse headache	27	21.1±4.7	19 (70.4)
Children Adolescents	5 22	20.8±4.4 21.6±4.8	3 (60.0) 16 (72.7)
Other headache on ≥ 15 days/month	64	18.3±3.6	24 (37.5)
Children Adolescents	22 42	18.6±3.3 18.2±3.9	4 (18.2) 20 (47.6)
**All headache** ^**1**^	2,032	4.2±4.4	615 (30.3)
Children Adolescents	963 1,069	4.5±3.8 4.0±4.8	318 (33.0) 297 (27.8)

^1^Includes unclassified headache

**Table 9:** Comparisons of headache types for measures of impaired participation (Kruskal-Wallis with post-hoc Dunn’s test)

**Lost time from school** (days/4 weeks)						
	***n***	**mean±SD; SE**	**TTH**	**UdH**	**pMOH**	**Other H15+**
Migraine	1,030	0.8±1.3;<0.1	**< 0.001**	**< 0.001**	**0.03**	> 0.05
TTH	395	0.6±1.3;0.1		> 0.05	**0.002**	**0.005**
UdH	483	0.6±1.1;<0.1			**0.001**	**0.003**
pMOH	27	4.6±6.3;1.2				> 0.05
Other H15+	64	1.8±3.1;0.4				
**Lost time from other activities** (days/4 weeks)						
Migraine	1,030	1.3±1.7;0.1	**< 0.001**	**< 0.001**	**< 0.001**	**< 0.001**
TTH	395	1.0±1.5;0.1		> 0.05	**< 0.001**	**< 0.001**
UdH	483	1.0±1.7;0.1			**< 0.001**	**< 0.001**
pMOH	27	10.2±10.0;1.9				> 0.05
Other H15+	64	5.2±6.4;0.8				
**Lost parental worktime** n^1^ (%) of parents of pupils with headache type reported to have lost time in preceding 4 weeks						
Migraine	334	32.4%	> 0.05	**0.03**	**0.002**	> 0.05
TTH	108	27.3%		> 0.05	**< 0.001**	> 0.05
UdH	121	25.1%			**< 0.001**	> 0.05
pMOH	19	70.4%				**0.04**
Other H15+	24	37.5%				

TTH: tension-type headache; UdH: undifferentiated headache; pMOH: probable medication-overuse headache; H15+: headache on ≥ 15 days/month; ^1^values of n in this analysis are numerators (all those with the headache type reporting lost parental worktime); denominators are all those with that headache type; significant p-values are emboldened

**Table 10:** Emotional impact and quality of life scores by headache type

Headache type	Pupils responding *n* ^1^	Score mean ± SD	Between-type significance (Kruskal-Wallis with post-hoc Dunn’s test)			
			TTH	UdH	pMOH	Other H15+
**Emotional impact** (0–18; higher scores adverse)						
Migraine	1,028	8.2±3.0	**0.001**	**< 0.001**	> 0.05	**0.03**
TTH	395	7.7±3.0		**0.02**	> 0.05	**< 0.001**
UdH	482	7.2±3.1			> 0.05	**< 0.001**
pMOH	26	7.7±3.5				> 0.05
Other H15+	64	9.3±3.5				
**Quality of life** (0–36; lower scores adverse)						
Migraine	1,030	18.5±6.9	**< 0.001**	**< 0.001**	> 0.05	> 0.05
TTH	395	20.6±6.2		0.05	**0.01**	**0.003**
UdH	483	21.3±6.2			**0.02**	**< 0.001**
pMOH	27	18.4±7.6				> 0.05
Other H15+	64	17.5±6.7				

TTH: tension-type headache; UdH: undifferentiated headache; pMOH: probable medication-overuse headache; headache on ≥ 15 days/month; ^1^values reflect that not all pupils responded to every enquiry; significant p-values are emboldened

## Discussion

For this study of headache-attributed burden among children and adolescents in Benin, the third such study in SSA following those in Ethiopia [13, 20] and Zambia [14, 15], we needed to address the inherent imprecision in epidemiological diagnosis of headache type among children and adolescents. In our earlier study reporting prevalences in Benin [1], our estimate for migraine (52.1%), based on reported symptoms, was unfeasibly high. The diagnosis of migraine was driven by ICHD criteria [6], which may not be appropriate for these age groups [21]. It was also dependent on the definition of UdH, which in the diagnostic methodology of these studies takes precedence over migraine and TTH [3]. Here we present revised estimates based on modified criteria for UdH, which increased the estimated prevalence of UdH from 8.4% [1] to 21.0%, but reduced the estimated prevalence of migraine only to 44.9%. Two findings in particular – the higher apparent prevalence of migraine among children than among adolescents, and the reverse for UdH – suggest either that symptoms were unreliably reported or that they are not, in these age groups, reliably diagnostic [21]. The drivers of diagnoses of migraine were the high proportions of those with headache who reported moderate or severe intensity (79.7%), throbbing character (68.2%), aggravation by routine activity (72.6%) or phonophobia (87.8%), while only 36.5% reported usual durations of > 2 h [1].

Our redefinition of UdH to include moderate headache, but retaining the crucial characteristic of very short duration (< 1 h), had been proposed in a study we reported from Georgia [9], and implemented in studies from Nepal [11] and Zambia [15]. The principal effect of this modification is that participants with moderate headache with migrainous features but duration < 1 h are classified as UdH rather than probable migraine. In reporting the Zambia findings, we recognised that this modification was not the full solution to the difficulty of defining UdH [15]. Evolving as the brain matures [2], UdH is not biologically defined (at least according to our present understanding), so diagnostic ambiguities – at least in epidemiological studies – are and will remain inevitable.

Most estimates of the elements of burden nevertheless clearly distinguished migraine from other episodic headaches (TTH and UdH). And it should be emphasised that our estimates of most elements of burden are not invalidated even if diagnostically misattributed: they exist, and are correctly *headache*-attributed. The exception is lost-health estimates derived from DWs used in GBD, which are diagnosis-specific. These estimates are anyway questionable, because the DWs were based on lay descriptions of the health loss associated with the ictal state of each headache type [18], which were not formulated with children or adolescents in mind.

With these caveats identified, we discuss the various elements of burden now.

The symptom burden was expressed in headache of moderate intensity occurring on an average of 4.2 days/4 weeks, but with short mean duration (2.8 h). All distributions were skewed by H15+. Of the episodic headaches, migraine was the most intense, most frequent and longest lasting, with pTIS of 2.3%. Among participants with H15+, with much higher frequencies and moderate to severe intensity, estimated pTIS was about 12%, a very large proportion of time. Not all headache episodes were medicated, probably because so many were of very short duration, although participants with pMOH used acute medication on 18.7 days/month, a worrying health risk in these young people.

Also of major concern in young people is lost school time, and impaired participation more generally: lost time from day-to-day activities, including play and social activities, which are also important among these age groups. All headache, among those affected, accounted for a material estimated loss (based on 4-week recall) of 4.0% of all school time, migraine > TTH, while pMOH and other H15 + were reportedly the cause of about a quarter of school time lost. We also assessed lost school time by reference to the more objective record of absence yesterday due to HY, an additional measure in the standardised methodology recognising that recall over 4 weeks is subject to error [3, 22]. With some variation between headache types, and TTH an outlier for no obvious reason, the two assessments matched for all headache. Predicted absence (from prevalence and reported frequency of headache-attributed absence) was 18.8%, while actual recorded absences were 15.8%, yielding a rather lower estimated loss of 3.3% of all school time among those with headache. However, in this latter estimate, lost days immediately preceding weekends or school holidays (> 20% of the total) would not have been counted. Days of limited activity were more than lost school days, remarkably so for pMOH and other H15+, but these should not be construed as wholly lost days.

Impact on parents was also material. Here, almost one in three (30.3%) parents lost time from their own work. For most headache types, parents of children were more likely to lose time than parents of adolescents, probably indicative of increasing independence somewhat offset by increasing prevalence among adolescents.

Because the emotional impact and QoL scores lack intuitive meaning, they are difficult to interpret. Neither showed much difference in impact between TTH and UdH, but, on each score, migraine had significantly greater impact than both, while H15 + had numerically but not significantly greater impact than migraine.

Our principal purpose in analysing between-type significances, for the measures of impaired participation in Table 9 and for emotional impact and QoL scores in Table 10, was to determine how well the diagnostic categories were distinguished from each other, including UdH according to the modified definition. On all measures, migraine was clearly distinguished (greater burden) from TTH and UdH, whereas only the emotional impact and QoL scores distinguished TTH (greater impact) from UdH. In other words, despite the diagnostic uncertainty, pupils classified as having migraine were reporting a headache measurably and consistently more burdensome than those classified as having TTH or UdH. Analyses such as these applied to much larger population- (not patient-) based datasets [23] may guide the development of more coherent diagnostic categorisation among the group with short-duration headache, perhaps considering all those with duration < 2 h (the current ICHD threshold for migraine in children [6]) rather than < 1 h.

The only study with comparable headache-attributed burden analyses (i.e., with the modified criteria for UdH) is ours from Zambia [15], also in SSA. There are many similarities. The two studies yielded similar, unfeasibly high estimates of migraine prevalence with the conventional definition of UdH (Zambia 51.6% [14], Benin 52.1% [1]), both reduced to a limited extent by modifying the criteria for UdH (to 42.9% [15] and 44.9% respectively). Burden estimates built upon the revised prevalence estimates were broadly similar, although symptom burden in Zambia (moderate-intensity headache, average frequency of 2.9 days/4 weeks, mean duration of 2.3 h, pTIS 1.2% [15]) was rather less than in Benin. Medication days were fewer than headache days in both countries. Days lost from school, estimated from HY data, were 13.8% in Zambia [15], equating (by multiplying by 1-day prevalence of 22.2% [1]) to 3.1% of all school time (*versus* 3.9% in Benin). More parents in Benin (30.3%) lost time than in Zambia (15.9% [15]), but in each case there were higher losses among parents of children.

Limitations of this study obviously included the diagnostic uncertainties, already fully discussed. Others were identified previously [1]. There were the general limitations of all surveys among young people arising from necessary dependence on their understanding and recall, the latter mitigated to an extent by additional enquiry into HY. A problem was the very high level of absenteeism (73%) – not unusual, despite free education in Benin [24] – which undermined the careful sampling procedure intended to ensure representativeness [1]. Absenteeism is both casual and permanent (drop-outs), with, in SSA generally, cultural, socioeconomic and behavioural determinants [25, 26]: cultural practices, such as puberty rites, may enforce seclusion of pubertal girls; one girl in ten in SSA does not attend school during her menstrual period, exacerbated in Benin by the lack, in many schools, of latrines and water points [27]; parents who are themselves poorly educated may see little value in educating their children, while older children put to income-generating work bring needed economic benefit; pupils abscond from classes without their parents’ knowledge, motivated by bullying, dislike of teachers or desire to impress friends, or through laziness, moodiness or boredom [25]. Also in Benin, teacher absenteeism has been identified as a factor [28]. Biases in our findings that might result from these highly diverse causes are indeterminable, although it should be noted that the sample remained reasonably well balanced for gender, and precisely balanced for urbanisation. Headache, if a cause, would not have been a significant factor within such a high level of absenteeism. Secondary headaches, including those due to malaria and other infections common in SSA, were unlikely to be reported as short-duration episodes (almost two thirds [63.5%] were described as usually lasting ≤ 2 h), while all participating pupils were well enough to be in class. Any secondary headaches that did occur would most likely have been among the 2.6% classified as other H15+. The strengths of the study lay in the tested and validated methodology [2], adequate sample size (N=2,295) [22] and high participating proportion (92.2%).

## Conclusions

Headache, prevalent among children and adolescents in Benin, is associated with burdens of lost health and impaired participation, the latter including lost school time. This, potentially, has lifelong negative impact. These burdens are reliably and robustly attributed to *headache*, but attribution to specific headache types is less certain because of unresolved difficulties of diagnosis in the many young people with short-duration headache. A solution to this problem may come from interrogation of large population-based datasets [23].

## Data Availability

The data are held on file at University of Mersin and the analytical subset also at Norwegian University of Science and Technology. Once analysis and publications are completed, they will be freely available for non-commercial purposes to any person requesting access in accordance with the general policy of the Global Campaign against Headache.

## Abbreviations

CI: Confidence interval
DW: Disability weight
GBD: Global Burden of Disease (study)
HARDSHIP: Headache-Attributed Restriction, Disability, Social Handicap and Impaired Participation (questionnaire)
HY: Headache yesterday
ICHD: International Classification of Headache Disorders
LTB: *Lifting The Burden*
MOH: Medication-overuse headache
pMOH: Probable MOH
pTIS: Proportion of time in ictal state
QoL: Quality of life
SD: Standard deviation
SE: Standard error (of the mean)
SSA: Sub-Saharan Africa
TTH: Tension-type headache
UdH: Undifferentiated headache
